# Immunoproteomic analysis of *Trichinella spiralis* and *Trichinella britovi* excretory-secretory muscle larvae proteins recognized by sera from humans infected with *Trichinella*

**DOI:** 10.1371/journal.pone.0241918

**Published:** 2020-11-05

**Authors:** Sylwia Grzelak, Anna Stachyra, Jerzy Stefaniak, Karolina Mrówka, Bożena Moskwa, Justyna Bień-Kalinowska

**Affiliations:** 1 Witold Stefański Institute of Parasitology, Polish Academy of Sciences, Warsaw, Poland; 2 Department and Clinic of Tropical and Parasitic Diseases, University of Medical Sciences, Poznań, Poland; Universita degli studi della Campania, ITALY

## Abstract

The present study compares the immunogenic patterns of muscle larvae excretory-secretory proteins (ML E-S) from *T*. *spiralis* and *T*. *britovi* recognized by *Trichinella*-infected human sera. Samples were analyzed using two-dimensional electrophoresis (2-DE) coupled with 2D-immunoblot and liquid chromatography-tandem mass spectrometry LC-MS/MS analysis, two ELISA procedures and a confirmatory 1D-immunoblot test. Sera were obtained from nine patients with a history of ingestion of raw or undercooked meat who presented typical clinical manifestations of trichinellosis and from eleven healthy people. Specific anti-*Trichinella* IgG antibodies were detected in all samples tested with the Home-ELISA kits, but in only four samples for the commercially-available kit. The 1D-immunoblot results indicated that all nine serum samples were positive for *T*. *spiralis* ML E-S antigens, expressed as the presence of specific bands. In contrast, eight of the serum samples with *T*. *britovi* E-S ML antigens were positive, with one serum sample taken from a patient at 33dpi (days post infection) being negative. To identify immunoreactive proteins that are specifically recognized by host antibodies, both species of ML E-S proteins were subjected to 2D-immunoblotting with human serum taken at 49 dpi. The sera recognized 22 protein spots for *T*. *spiralis* and 18 for *T*. *britovi* in 2D-immunoblot analysis. Their molecular weights (MW) ranged from 50 to 60 kDa. LC-MS/MS analysis identified both common and specifically-recognized immunoreactive proteins: transmembrane serine protease 9, serine protease, antigen targeted by protective antibodies and Actin-1 partial were shared for both *Trichinella* species; hypothetical protein T01_7775 and P49 antigen, partial those specific to *T*. *spiralis*; deoxyribonuclease-2-alpha and hypothetical protein T03_17187/T12_7360 were specific to *T*. *britovi*. Our results demonstrate the value of 2-DE and 2D-immunblot as versatile tools for pinpointing factors contributing to the parasite-host relationship by comparing the secretomes of different *Trichinella* species.

## Introduction

Trichinellosis is a zoonotic parasitic disease caused by nematodes of the genus *Trichinella* [1]. Humans can be infected by the ingestion of raw or undercooked meat products containing live *Trichinella* larvae [2]. The complete life cycle of *Trichinella* occurs in a single host, including all stages of the adult worms (Ad), newborn larvae (NBL) and muscle larvae (ML). During all developmental stages, *Trichinella* express many immunodominant antigens that have the ability to elicit a significant immune response. In *T*. *spiralis* and *T*. *britovi* muscle larvae (ML), the excretory-secretory (E-S) proteins are produced mainly by the excretory granules of the stichosome and the cuticles (membrane proteins); these are directly exposed to the host immune system and are the main target antigens which induce immune responses [3]. Most human infections and deaths caused by trichinellosis globally have been attributed to *T*. *spiralis*. However, a number of other encapsulating and non-encapsulating species can cause human infections, including *T*. *britovi*, *T*. *nativa*, *T*. *pseudospiralis* and *T*. *murelli* [4–8].

Trichinellosis may represent a serious risk for human health, and in some cases, can have a fatal outcome [9]; however, the patient may remain asymptomatic if only a few live *Trichinella* larvae are ingested. The condition is often manifested by a range of symptoms, including gastroenteritis, fever, headache, myalgia, malaise, facial edema, subungual or conjunctival hemorrhages; in addition, increased eosinophil and leukocyte counts, and elevated muscle enzyme levels are observed in laboratory tests [10].

Although clinical differences have been observed between infections by different *Trichinella* species, it has not been possible to make clear links between symptoms and a particular species due to the presence of confounding factors, such as the number of infective larvae ingested by a patient [11]. Nevertheless, the clinical features observed during human infection with *T*. *spiralis* appear to be different from those caused by *T*. *britovi*, with *T*. *spiralis* infections being characterized by a longer duration of parasite-specific IgG and increased creatine phosphokinase (CPK) levels compared to those of *T*. *britovi*. Additionally, patients infected with *T*. *spiralis* typically present with a more severe intestinal symptomatology than those infected with *T*. *britovi*, possibly due to the fact that *T*. *britovi* females are less prolific in the production of new born larvae (NBL) [12]. Infections caused by other species, such as *T*. *murrelli*, seem to be more likely to provoke skin reactions and facial edema [13], whereas those triggered by non-encapsulated *T*. *pseudospiralis* appear to provoke longer-lasting symptoms [14].

The diagnosis of human trichinellosis is complicated by the nonspecific nature of its symptoms [10]. Currently, a combination of patient epidemiological history, laboratory testing including specific enzyme-linked immunosorbent assay (ELISA) for the detection of specific *Trichinella* antibodies, and detection of larvae in a muscle biopsy, are required for a definitive diagnosis of human trichinellosis. Muscle biopsy is an invasive and painful procedure for the patient, and positive results are often not obtained, even when infection is present [1,11].

Unfortunately, existing ELISA-based serological tests based on the *Trichinella* excretory-secretory antigens of muscle larvae tend to be unreliable [15–17]. Therefore, to improve *Trichinella* diagnosis in humans, ELISA should be paired with a confirmatory test such as immunoblot. This approach offers higher specificity by allowing visualization of the *Trichinella-*specific proteins which react with host antibodies. In addition, a more exhaustive approach for comparing immunogenic proteins is based on the combination of classical 1D-immunoblot analysis with more effective proteomic techniques, such 2-DE electrophoresis coupled with 2D-immunoblot and mass spectrometry. This approach allows more accurate identification of specific proteins recognized by human *Trichinella*-infected sera, thus improving serodiagnosis and facilitating the development of a vaccine against the parasite.

Therefore, the purpose of the present study was to compare the immunogenic patterns of the ML E-S proteins of *T*. *spiralis* and *T*. *britovi* and select immunoreactive proteins recognized by human *Trichinella*-infected sera by a combination of two-dimensional electrophoresis (2-DE) with 2D-immunoblot and liquid chromatography-tandem mass spectrometry LC-MS/MS analysis. The antibody response was evaluated by two ELISA procedures with a confirmatory 1D-immunoblot test.

## Materials and methods

### Ethics approval and consent to participate

All experimental procedures used in the present study had been pre-approved by the First Local Ethical Committee for Scientific Experiments on Animals in Warsaw, Poland (resolution no.: 020/2016, 23 March 2016). Written informed consent was obtained from all patients hospitalized in the Department and Clinic of Tropical and Parasitic Diseases, University of Medical Sciences in Poznań.

### Patient sera

Human serum samples with trichinellosis were collected from nine patients during an outbreak that occurred in December/January 2002/2003 and March 2004 in the Wielkopolskie Voivodship, Western Poland. The patients were hospitalized in the Department and Clinic of Tropical and Parasitic Diseases, University of Medical Sciences in Poznań. The diagnosis of trichinellosis was confirmed for all of these patients by the presence of a high fever associated with headache, nausea, diarrhea, facial edema and a history of ingestion of raw or undercooked meat (sausage with pork and wild boar meat) containing *Trichinella* spp. infective muscle larvae. The first clinical symptoms of infection were observed between 9 and 35 days following consumption of infective meat. Control negative serum samples were obtained from 11 healthy adult volunteers who were free from any intestinal parasitic infection. All serum samples were stored at -70 ^o^C for use in ELISA and immunoblot analysis. Muscle biopsy was not performed.

### Parasites and ML E-S preparation

The tested ML E-S antigens were obtained from *Trichinella spiralis* (strain ISS-003) and *T*. *britovi* (strain ISS-002) ML (Istituto Superiore di Sanita, The International *Trichinella* Reference Centre). These were subjected to several passages in female CH3/W mice at the W. Stefanski Institute of Parasitology, PAN. Briefly, the mice were infected with 500 ML of *T*. *spiralis* and *T*. *britovi*; these were recovered on day 42 post infection (dpi) by HCl-pepsin digestion. ML E-S antigens were obtained as described previously [16,18,19]. Briefly, the obtained muscle larvae were washed with RPMI-1640 and resuspended at 5000 ML/ml in RPMI-1640 supplemented with 20 mM HEPES, 200 mM L-glutamine, 100 mM Na-pyruvate and 100 units of both penicillin and streptomycin. The obtained ML were then incubated in a T-75 culture flask in 5% CO2 at 37°C for 18 hours. The culture supernatants were then filtered through 0.22μm membranes to obtain E-S proteins. The filtered supernatant was then lyophilized. The ML E-S protein concentration was determined using a NanoDrop spectrophotometer.

### ELISA

All serum samples (cases and controls) were tested for the presence of anti-*Trichinella* IgG antibodies using two procedures: commercially-available *T*. *spiralis* IgG-ELISA kit (NovaTec, Immunodiagnostica GMBH, TRIG0480) and Home-ELISA described by Moskwa et al., (2006, 2009) [16,18], and Gondek et al., (2018) [20].

Briefly, in Procedure 1, the human serum samples were tested using a commercial *T*. *spiralis* IgG-ELISA kit (NovaTec). The procedure for E-S antigen preparation in the NovaTec kit is not available. In Procedure 2, *T*. *spiralis* E-S ML antigens prepared in the W. Stefański Institute of Parasitology, Polish Academy of Sciences were tested with Home-ELISA. The cut-off for Home-ELISA was calculated based on the mean OD plus three standard deviations (S.D.) of 10 serum samples of healthy patients.

### SDS-PAGE and 1D-immunoblot analysis

Briefly, 20 μg of each of ML E-S antigens were dissolved in 2 x Laemmli sample buffer (Sigma) and boiled for five minutes as reducing condition and then were subjected to SDS-PAGE (4% stacking gels and 12% resolving gels). Electrophoresis was performed in Mini-PROTEAN Tetra Cell electrophoresis chamber (BioRad, USA) at 200 V for approximately 50 minutes. After electrophoresis, gels were silver-stained using PlusOne Silver Staining Kit (GE Healthcare) in accordance with the manufacturer’s instructions.

The unstained gels were transferred onto Immuno-Blot polyvinylidene fluoride (PVDF) membranes (BioRad) by a wet transfer system (BioRad, USA) at 95 V for one hour in cool conditions. The transferred membranes were then blocked in buffer with 5% milk in 20 mM Tris-HCl, 0.9% NaCl, pH 9.0 for one hour at room temperature. Following this, the membranes were cut into regular strips, which were subsequently incubated with human serum samples diluted 1:100 in phosphate-buffered saline; the incubation was performed overnight at 4°C with 5% skimmed milk solution. Incubation with the secondary antibody, anti-Human IgG (whole molecule) antibody produced in rabbit (Sigma-Aldrich, Louis, USA) diluted 1:10 000 in PBS with 5% skimmed milk, was then performed for one hour at room temperature. Any immunoreactions were visualized by incubating all the separate strips with a substrate solution containing SIGMAFAST™ 3,3′-Diaminobenzidine (Sigma-Aldrich, Louis, USA) for at least 10 minutes.

### 2-DE and 2D-immunoblot analysis

Two replicates of *T*. *spiralis* and *T*. *britovi* ML E-S protein samples were run in parallel on immobilized pH gradient IPG strips (RioRad, Hercules, USA). Briefly, 100 μg of previously prepared ML E-S protein of both *Trichinella* species were purified with the 2-D Clean-Up Kit (GE Healthcare, New Jersey, USA) in accordance with the manufacturer’s protocol. After the final centrifugation step, the protein pellets were rehydrated overnight in 250 μl of 2-D Starter Kit Rehydratation/Sample Buffer (BioRad, Hercules, USA) and loaded onto 7 cm pH 3–10 IPG strips (BioRad, Hercules, USA). The first dimension, i.e. isoelectric focusing (IEF), was performed with a Protean IEF Cell (BioRad) device at 20°C according to Grzelak et al. (2018) [19]. After focusing, the strips were submitted for two steps of equilibration in equilibration buffers, the first for 25 minutes in ReadyPrep 2-D starter Kit Equilibration Buffer I, containing DTT (BioRad, USA), and the second for 25 minutes in ReadyPrep 2-D Starter Kit Equilibration Buffer II containing iodoacetamide (BioRad, USA) instead of DTT. The second dimension SDS-PAGE was run using 12% acrylamide separating and 4% polyacrylamide stacking gels in the Mini-PROTEAN Tetra Cell electrophoresis chamber (BioRad, USA) at 200 V for approximately 55 minutes.

After 2-D electrophoresis, gels with 100 μg of each sample were silver-stained using PlusOne Silver Staining Kit (GE Healthcare) in accordance with the manufacturer’s protocol or used without staining for 2-DE immunoblotting. The obtained gels were scanned with a ChemiDoc MP system (BioRad, USA) and analyzed in Image Lab 5.2.1. software (BioRad, USA). At the same time, the proteins from the unstained gels were transferred onto Immun-Blot polyvinylidene fluoride (PVDF) membrane (BioRad) by a wet transfer system (BioRad, USA) at 95 V for one hour in cool conditions. The PVDF membranes with *T*. *spiralis* and *T*. *britovi* ML E-S proteins were blocked in Pierce Protein-Free T20 (TBS) Blocking Buffer (ThermoFisher Scientific) for one hour at room temperature. Following this, the PVDF membranes were incubated overnight at 4°C with a single selected human serum sample (sample no. 5) diluted 1:100. The membranes were then incubated with anti-Human rabbit IgG (whole molecule) antibody (Sigma-Aldrich, Louis, USA) diluted 1: 10 000 for one hour at room temperature. Uninfected sera was used as a parallel negative control.

The immunoreactive proteins were visualized on a film with the use of the Super Signal West Pico Chemiluminescent Substrate (ThermoFisher Scientific, Walthman, USA). Reproducibility of the immune recognition was verified by repeating the immunoblot at least two times.

### LC-MS/MS

The samples were subjected to standard trypsin digestion, during which the proteins were reduced with 10 mM DTT for 30 minutes at 56°C, alkylated with iodoacetamide in darkness for 45 minutes at room temperature and digested overnight with 10 ng/μl trypsin. The resulting peptide mixtures were concentrated and desalted on a RP-C18 pre-column (Waters), and further peptide separation was achieved on a nano-Ultra Performance Liquid Chromatography (UPLC) RP-C18 column (Waters, BEH130 C18 column, 75 μm i.d., 250 mm long) of a nanoACQUITY UPLC system, using a 45 min linear acetonitrile gradient. Column outlet was directly coupled to the Electrospray ionization (ESI) ion source of the Orbitrap Velos type mass spectrometer (Thermo Scientific, Waltham, USA), working in the regime of data dependent MS to MS/MS switch with HCD type peptide fragmentation. An electrospray voltage of 1.5 kV was used.

### Bioinformatics

Raw data files were pre-processed with Mascot Distiller software (version 2.6, MatrixScience, London, UK). The obtained peptide masses and fragmentation spectra were matched to the NCBInr database (147075655 sequences; 53900923684 residues), with a Nematoda filter (928973 sequences) using the Mascot search engine (MatrixScience, London, UK, Mascot Server 2.5). The following search parameters were applied: enzyme specificity was set to trypsin, peptide mass tolerance to ± 30 ppm and fragment mass tolerance to ± 0.1 Da. The protein mass was left as unrestricted, and mass values as monoisotopic with one missed cleavage being allowed. Alkylation of cysteine by carbamidomethylation as fixed and oxidation of methionine was set as a variable modification. Protein identification was performed using the Mascot search engine (MatrixScience), with the probability-based algorithm. An expected value threshold of 0.05 was used for analysis, which means that all peptide identifications had less than 1 in 20 chance of being a random match.

All proteins identified in the Mascot search were assigned to the UniProtKB database (https://www.uniprot.org/) and QuickGO (http://www.ebi.ac.uk/QuickGO/) and classified in gene ontology (GO) in accordance with its molecular function, biological process and cellular component information.

## Results

### ELISA

The anti-*Trichinella* IgG levels in the serum samples of patients with trichinellosis were determined using the two ELISA procedures (Table 1). The cut-off values for Procedure 1 and Procedure 2 were 1.4 and 0.27, respectively. Four human serum samples were found to be positive using the commercially-available *T*. *spiralis* IgG ELISA kit (NovaTec), but the OD values of all nine individual serum samples varied from 0.689 to 1.71 (Table 1); in contrast, all serum samples examined for the presence of anti-*Trichinella* IgG using Home-ELISA were positive, and the OD values varied from 0.287 to 0.587. Two negative sera (OD values of 0.198 and 0.215) were randomly-selected for further immunoblot analysis; the results are presented in Table 1. Both seropositive and borderline samples were tested by immunoblot to confirm the presence of anti-*Trichinella* antibodies.

**Table 1:** Detection of the anti-*Trichinella* IgG antibodies in sera from patients infected with *Trichinella* spp. by commercial ELISA and home ELISA.

Patients	Day of consumption of infective meat	Day of first clinical symptoms	Number of days from consumption of infective meat to the first symptoms/data of blood collection	Commercial ELISA OD (cut-off 1.4)	Home ELISA OD (cut-off 0.27)
Patient 1	01.03.2004	10.03.2004	9/33	1.183	**0.418**
Patient 2	02.03.2004	12.03.2004	10/27	0.868	**0.374**
Patient 3	01.01.2003	11.01.2003	10/36	**1.446**	**0.520**
Patient 4	25.12.2002	06.01.2003	12/44	**1.564**	**0.464**
Patient 5	25.12.2002	07.01.2003	13/44	0.796	**0.287**
Patient 6	23.12.2002	06.01.2003	14/49	**1.426**	**0.439**
Patient 7	23.12.2002	15.01.2003	23/48	0.689	**0.347**
Patient 8	27.12.2002	20.01.2003	24/43	**1.710**	**0.587**
Patient 9	25.12.2002	29.01.2003	35/45	1.361	**0.439**
Patient 10	Negative control	-	-	-	0.198
Patient 11	Negative control	-	-	-	0.215
Patient 12	Negative control	-	-	-	0,231
Patient 13	Negative control	-	-	-	0,130
Patient 14	Negative control	-	-	-	0,194
Patient 15	Negative control	-	-	-	0,137
Patient 16	Negative control	-	-	-	0,152
Patient 17	Negative control	-	-	-	0,123
Patient 18	Negative control	-	-	-	0,157
Patient 19	Negative control	-	-	-	0,112
Patient 20	Negative control	-	-	-	0,169

### Protein profiles and 1D-immunoblot reactivity of *Trichinella*-infected human sera with *T*. *spiralis* and *T*. *britovi* ML E-S

1-dimensional analysis based on *T*. *spiralis* and *T*. *britovi* ML E-S antigens were used to confirm the results obtained in ELISA and to detect *Trichinella* specific-proteins.

**Fig 1:**
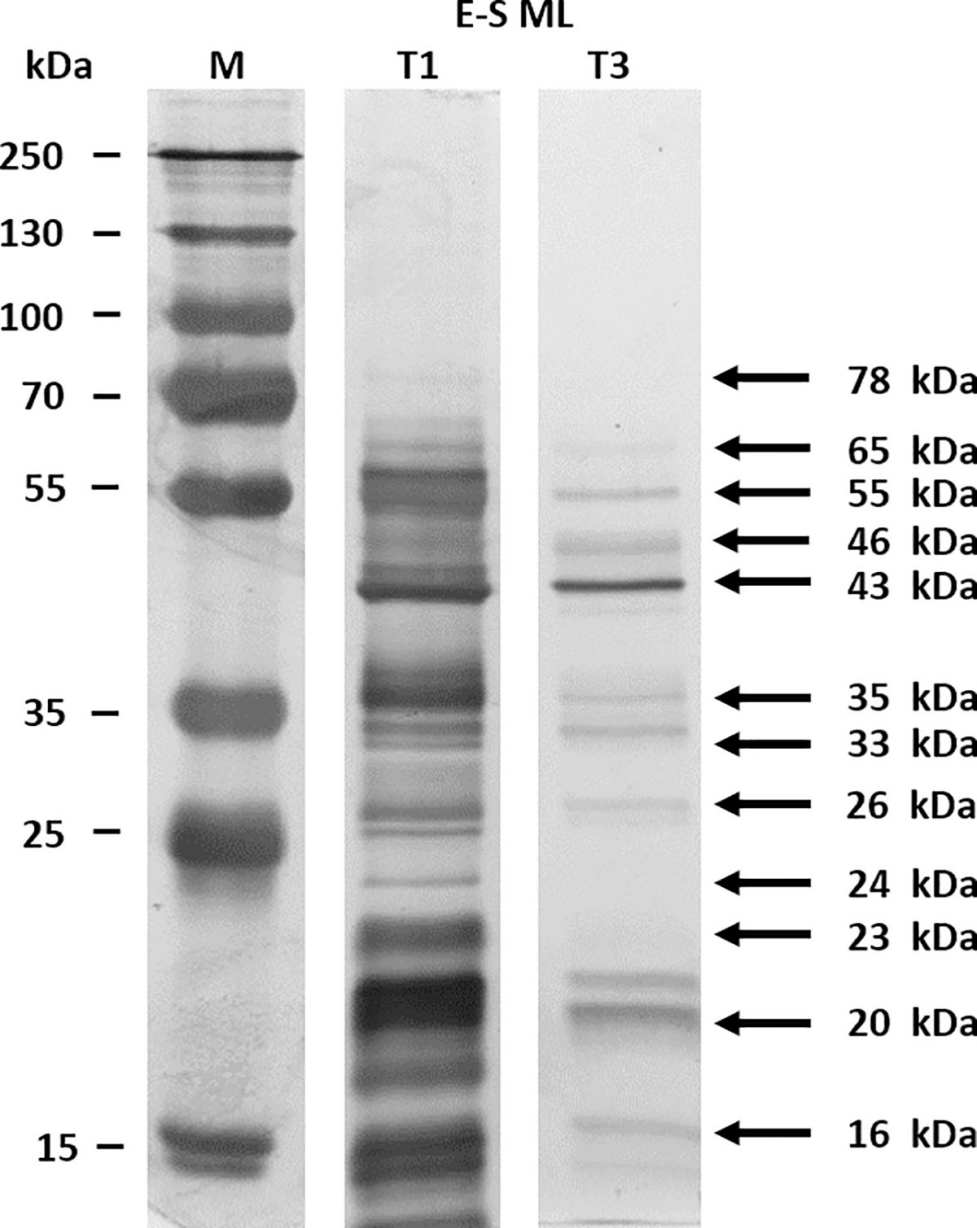
SDS-PAGE analysis of muscle larvae (ML) excretory-secretory proteins of *T*. *spiralis* (E-S T1) and *T*. *britovi* (E-S T3).

The electrophoretic profiles of *T*. *spiralis* and *T*. *britovi* ML E-S proteins revealed that the majority were in the range 15 to 100 kDa (Fig 1). Some differences were observed in the number and intensity of specific bands between both protein patterns: (i) more bands were observed in the protein patterns of *T*. *spiralis* than *T*. *britovi*; (ii) the 78 and 64 kDa bands in *T*. *spiralis* ML E-S were more intense than those in *T*. *britovi* ML E-S; (iii) the 43, 57, 33 and 22 kDa bands were of similar intensity (Fig 1).

**Fig 2:**
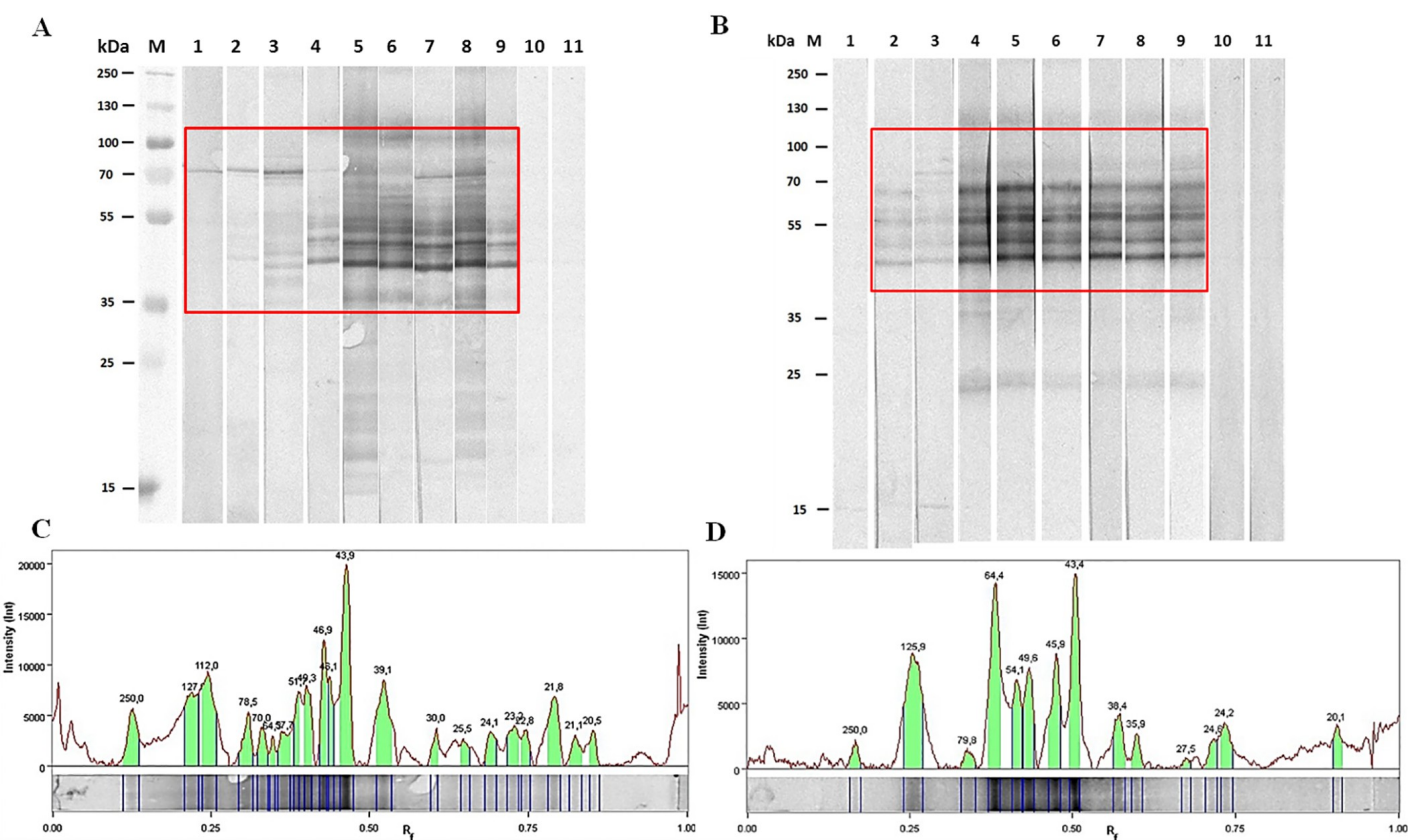
The immunoblot analysis of A) *T*. *spiralis* and B) *T*. *britovi* E-S ML proteins incubated with *Trichinella*-infected human sera samples (lanes 1–9), and the negative control samples (lanes 10 and 11). The red box indicates the area with the highest differences in the immunoblot patterns between *Trichinella* species. Signal intensity and relative migration values of *T*. *spiralis* (C) and *T*. *britovi* (D) ML E-S with *Trichinella*-infected sera (analysis of lane 6).

Sera from nine patients with trichinellosis reacted with the *T*. *spiralis* and *T*. *britovi* ML E-S; however, they displayed different reactivity patterns with regard to the presence, or absence, of specific bands and their intensity (Fig 2A and 2B). Representative patterns, demonstrating the differences in a signal intensity and relative migration values of *T*. *spiralis* and *T*. *britovi* ML E-S reactive proteins with representative human serum samples at 49 dpi (days post infection), are given in Fig 2C and 2D.

The 1D-immunoblot results indicated that all nine serum samples were positive for *T*. *spiralis* ML E-S antigens, expressed as the presence of specific bands. In contrast, eight of the serum samples with *T*. *britovi* ML E-S antigens were positive, with one serum sample taken from a patient at 33dpi being negative (Fig 2B, line 1).

The intensity of immune response varied between patients, and correlated with the first symptoms and numbers of days post infection (dpi). The sera from a patient taken 33 days after consuming of infected meat recognized only one intense protein band of 70 kDa when *T*. *spiralis* ML E-S antigens were used (Fig 2A, lane 1), but no bands when the *T*. *britovi* E-S ML antigens were used (Fig 2B, lane 1). This specific 70 kDa band was also observed in both *Trichinella* protein patterns when serum samples from patients at 27 to 49 dpi were used, together with other high intensity bands in the region 43 to 55 kDa (Fig 2, lanes 2–8). Additionally, a specific protein band around 120 kDa was observed when *T*. *spiralis* and *T*. *britivi* ML E-S were blotted with sera from patients at 44–49 dpi (Fig 2A and 2B, lanes 4–9). Sera from healthy persons did not recognize *Trichinella* protein bands.

### 2DE analysis and immunoreactive proteins of *T*. *spiralis* and *T*. *britovi* E-S ML recognized by *Trichinella*-infected human sera

**Fig 3:**
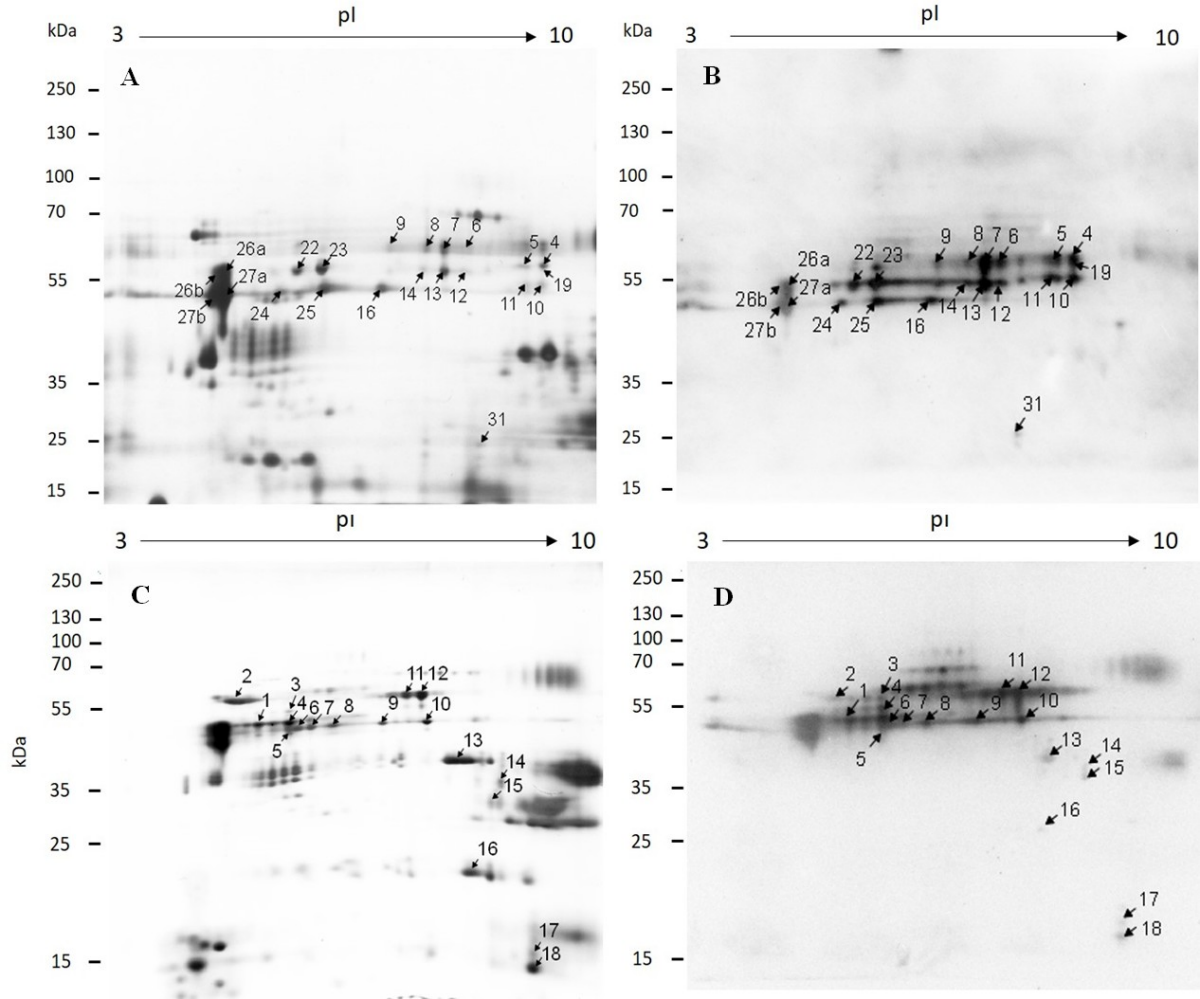
An image of 2-DE separations and immunoblot analysis of *T*. *spiralis* and *T*. *britovi* E-S ML proteins. **A-***T*. *spiralis***, C-***T*. *britovi*—2-DE gels were stained with silver stain; 2D-immunoblot of *T*. *spiralis* **(B)** and *T*. *britovi* **(D)** proteins were probed with infected human sera at 14 dpi. Matched spots selected for subsequent LC-MS/MS analysis are marked.

The purified *T*. *spiralis* and *T*. *britovi* ML E-S proteins were analyzed by two-dimensional electrophoresis (2-DE) coupled with protein identification by liquid chromatography-tandem mass spectrometry (LC-MS/MS) (Fig 3A and 3C). To identify immunoreactive proteins that are specifically recognized by host antibodies, both species of ML E-S proteins were subjected to two-dimensional (2-DE) immunoblotting with human serum taken at 49 dpi (Fig 2A and 2B, line 6). The serum sample was chosen based on the ELISA results and intensities of protein bands.

Fig 3A and 3C represent one of the three replicated silver-stained proteome gels used for further analysis. A total of approximately 150 spots were detected on the silver stained 2-DE gels, with pI varying from 3 to 10 and molecular weight from 25 to 70 kDa (Fig 3A and 3C)

Major protein spots for *T*. *spiralis* and *T*. *britovi* ML E-S were located between 35 to 60 kDa.

Approximately 31 *T*. *spiralis* ML E-S immunoreactive spots were positively recognized by *Trichinella*-infected human serum samples at 49 dpi. In the *T*. *spiralis* ML E-S proteome, 21 out of 22 marked protein spots with intensive chemiluminescence signal were found to migrate: their molecular weights ranged from 50 to 60 kDa (Fig 3B). One immunoreactive spot (no. 31) was located at a height of 25 kDa MW.

For *T*. *britovi* ML E-S, approximately 18 immunoreactive protein spots were recognized by antibodies in human serum sample at 49 dpi. Most of these proteins were located in the area between 50 to 60 kDa. Some of the individual immunogenic spots were found in the area between 40 to 50 kDa, and between 30 kDa and 18 kDa (Fig 3D). No protein reacted to uninfected swine sera.

### Identification of proteins by LC-MS/MS analysis

Immunoreactive spots were matched to the corresponding protein spots on silver-stained gels, and were selected for further LC-MS/MS identification. The LC-MS/MS identification identified common and species-specific proteins among two *Trichinella* species (Tables 2 and 3). Therefore, a total of 22 protein spots of *T*. *spiralis* ML E-S were selected for final identification; however, only 17 were successfully identified. In the *T*. *spiralis* ML E-S proteome, the immunoreactive protein spots were identified as transmembrane serine protease 9 (spots no. 4, 7, 10), serine protease (spot no. 5, 11, 12, 13, 14, 19), hypothetical protein T01_7775 (spot no. 31), P49 antigen, partial (spot no. 16, 25), antigen targeted by protective antibodies (spot no. 26 a, b; 27 a, b), Actin 1, partial (spot no. 16, 26b). Most of these spots were identified in multiple protein spots (Table 2, Fig 3B).

**Table 2:** Results of LC-MS/MS analysis of selected spots from *Trichinella spiralis* muscle larvae excretory-secretory proteins which reacted with *Trichinella*-infected human serum.

Spot	DB^a^	Accession	MS^b^	MP^c^	Seq^d^	SC^d^(%)^e^	emPAI^f^	Mr(kDa)/pI^g^	Description
**4**	NCBIprot	KRY34986.1	261	7	5	3%	0.20	127,86/6,42	Transmembrane protease serine 9
**5**	NCBIprot	ACA28932.1	153	4	3	5%	0.30	48,67/6,33	serine protease
**6**	Unidentified	-	-	-	-	-	-	-	-
**7**	NCBIprot	KRY34986.1	278	4	4	3%	0.11	127,86/6,42	Transmembrane protease serine 9
**8**	Unidentified	-	-	-	-	-	-	-	-
**9**	NCBIprot	ACA28932.1	147	3	3	5%	0.30	48,67/6,33	serine protease
**10**	NCBIprot	KRY34986.1	248	8	5	3%	0.14	127,86/6,42	Transmembrane protease serine 9
**11**	NCBIprot	ACA28932.1	204	5	4	7%	0.42	48,67/6,33	serine protease
**12**	NCBIprot	ACA28932.1	147	4	3	5%	0.31	48,67/6,33	serine protease
**13**	NCBIprot	ACA28932.1	165	4	3	5%	0.30	48,67/6,33	serine protease
**14**	NCBIprot	ACA28932.1	159	4	3	5%	0.31	48,67/6,33	serine protease
**16**	NCBIprot	AAA30328.1	404	7	6	21%	1.34	34,54/5,23	P49 antigen, partial
	NCBIprot	KRY05075.1	87	1	1	5%	0.24	19,10/5,12	Actin-1, partial
**19**	NCBIprot	ACA28932.1	211	5	4	7%	0.42	48,67/6,33	serine protease
**22–24**	Unidentified	-	-	-	-	-	-	-	-
**25**	NCBIprot	AAA30328.1	308	5	5	17%	0.69	34,54/5,23	P49 antigen, partial
**26a**	NCBIprot	AAA20539.1	106	3	2	5%	0.50	31,70/4,76	antigen targeted by protective antibodies
**26b**	NCBIprot	AAA20539.1	108	2	2	7%	0.31	31,70/4,76	antigen targeted by protective antibodies
	NCBIprot	KRY05075.1	50	1	1	5%	0.25	19,10/5,12	Actin-1, partial
**27a**	NCBIprot	AAA20539.1	205	4	3	9%	0.49	31,70/4,76	antigen targeted by protective antibodies
**27b**	NCBIprot	AAA20539.1	202	4	4	12%	0.73	31,70/4,76	antigen targeted by protective antibodies
**31**	NCBIprot	KRY36588.1	113	2	2	15%	0.82	15,81/6,28	hypothetical protein T01_7775

^a^DB-database

^b^MS-mascot score

^c^MP-matched peptide

^d^Seq-sequences

^e^SC-sequence coverage

^f^emPAI-exponentially modified protein abundance index

^g^Mr/pI-experimental nominal mass and isoelectric point.

**Table 3:** Results of LC-MS/MS analysis of selected spots from *Trichinella britovi* muscle larvae excretory-secretory proteins which reacted with *Trichinella*-infected human serum.

Spot	DB^a^	Accession	MS^b^	MP^c^	Seq^d^	SC(%)^e^	emPAI^f^	Mr(kDa)/pI^g^	Description
**1**	NCBIprot	AAA20539.1	58	2	1	4%	0.14	31,70/4,76	antigen targeted by protective antibodies
**2–3**	Unidentified	-	**-**	**-**	**-**	**-**	**-**	**-**	-
**4**	NCBIprot	KRY50178.1	329	6	6	13%	0.91	46,78/5,44	hypothetical protein T03_17187
**5**	NCBIprot	KRY12204.1	193	4	4	8%	0.37	48,54/5,68	hypothetical protein T12_7360, partial
**6**	NCBIprot	KRY50178.1	352	8	6	13%	1.37	46,78/5,44	hypothetical protein T03_17187
	NCBIprot	KRX47308.1	214	6	4	4%	0.27	107,15/6,52	Deoxyribonuclease-2-alpha
**7**	NCBIprot	KRY12204.1	363	8	6	12%	1.07	48,54/5,68	hypothetical protein T12_7360, partial
**8**	NCBIprot	KRX47308.1	210	3	3	3%	0.13	107,15/6,52	Deoxyribonuclease-2-alpha
**9**	NCBIprot	KRX47308.1	198	6	3	3%	0.17	107,15/6,52	Deoxyribonuclease-2-alpha
**10**	NCBIprot	KRX47308.1	236	4	4	4%	0.18	107,15/6,52	Deoxyribonuclease-2-alpha
**11**	NCBIprot	ACA28932.1	156	4	3	5%	0.30	48,67/6,33	serine protease
**12**	NCBIprot	ACA28932.1	155	5	3	5%	0.30	48,67/6,33	serine protease
**13**	NCBIprot	KRX20911.1	99	2	2	2%	0.13	73,14/8,82	Transmembrane protease serine 9
**14**	NCBIprot	KRY21930.1	200	3	3	2%	0.14	113,74/8,48	Transmembrane protease serine 9
**15**	NCBIprot	KRY05075.1	51	1	1	5%	0.25	19,10/5,12	Actin-1, partial
**16**	NCBIprot	KRY13097.1	51	1	1	2%	0.12	41,17/6,09	hypothetical protein T12_3826, partial
**17**	Unidentified	-	-	-	-	-	-	-	-
**18**	NCBIprot	KRY16814.1	77	1	1	5%	0.25	20,090/8,56	hypothetical protein T12_16967

^a^DB-database

^b^MS-mascot score

^c^MP-matched peptide

^d^Seq-sequences

^e^SC-sequence coverage

^f^emPAI-exponentially modified protein abundance index

^g^Mr/pI-experimental nominal mass and isoelectric point.

Of a total of 18 protein spots of *T*. *britovi* ML E-S subjected to LC-MS/MS analysis, 15 were successfully identified as antigen targeted by protective antibodies (spot no. 1), deoxyribonuclease-2-alpha (DNase II) (spots 6, 8, 9, 10), serine protease (spots 11, 12) transmembrane serine protease 9 from (spots13, 14), Actin-1, partial (spot no. 15), and some spots were identified as hypothetical proteins (spots 4, 5, 6, 7, 16, 18) (Table 3, Fig 3D).

### GO analysis (common and specific proteins)

The gene ontology (GO) database was used to segregate the antigenic proteins of two *Trichinella* species according to their molecular function, cellular component and biological process (Tables 4 and 5).

**Table 4:** GO categories of *T*. *spiralis* immunoreactive E-S ML proteins.

Protein name	Gene ontology annotations		
	biological process	cellular component	molecular function
**hypothetical protein T01_7775**	-	-	ribonucleoprotein*
**transmembrane protease serine 9**	-	integral component of membrane	serine-type endopeptidase activity
**antigen targeted by protective antibodies**	proteolysis**	-	serine-type endopeptidase activity**
**actin-1 (Fragment)**	-	-	ATP-binding***
**p49 antigen (Fragment)**	-	-	deoxyribonuclease II activity
**serine protease**	-	-	serine-type endopeptidase activity

BLAST analysis of sequence identity with different *Trichinella* spp. proteins

*77,4% identity with U3 small nucleolar ribonucleoprotein IMP4

**BLAST—98,9% identity with peptidase S1 domain-containing protein

***BLAST—Actin domain family.

**Table 5:** GO categories of *T*. *britovi* immunoreactive E-S ML proteins.

Protein name	Gene Ontology annotations		
	biological process	cellular component	molecular function
**hypothetical protein T03_17187**	-	-	-
**hypothetical protein T12_7360 (fragment)**	-	-	-
**antigen targeted by protective antibodies**	proteolysis*	-	serine-type endopeptidase activity*
**hypothetical protein T12_16967**	-	integral component of membrane	-
**transmembrane protease serine 9**	-	integral component of membrane	serine-type endopeptidase activity
**hypothetical protein T12_3826 (fragment)**	-	-	-
**actin-1 (Fragment)**	-	-	ATP-binding**
**transmembrane protease serine 9**	-	integral component of membrane	serine-type endopeptidase activity
**deoxyribonuclease-2-alpha**	-	-	deoxyribonuclease II activity
**serine protease**	-	-	serine-type endopeptidase activity

BLAST analysis of sequence identity with different *Trichinella* spp. proteins

*98,9% identity with peptidase S1 domain-containing protein

**Actin domain family.

## Discussion

In humans, trichinellosis is typically diagnosed using serological methods, with ELISA being the most commonly-used screening test. International Commission on Trichinellosis (ICT) recommends that ELISA-based serological testing methods should be based on ML E-S antigens obtained from *Trichinella* maintained *in vitro*. This antigen preparation contains a group of immunodominant, structurally-related glycoproteins that are recognized by animals and humans infected with *T*. *spiralis*, or any of the other currently-known species of *Trichinella* [18,21,22]. However, the main disadvantage of using ELISA based on ML E-S antigens is that a high rate of false negative results is typically observed during the early stage of infection, indicating that ML E-S antigens are stage-specific and are not recognized by antibodies during intestinal phase of infection. Thus, the immunoblot to confirm ELISA- positive results is recommended [15–17,22,23].

The combination of two-dimensional electrophoresis (2-DE) with mass spectrometry LC-MS/MS analysis is an effective approach which was used in the present study for high resolution analysis of ML E-S proteins from two *Trichinella* species. Supplementing these techniques with 2D-immunoblot using *Trichinella*-infected human sera allowed us to identify both species-specific and common proteins which induce host immune responses during infection and could therefore be used for improved serodiagnosis of trichinellosis or for vaccine development.

Firstly, to select sera for immunoproteomic analysis, all serum samples were tested by two ELISA protocols; the samples taken from nine patients with a history of ingestion of raw or undercooked meat and who presented with typical clinical manifestations of trichinellosis. The specific anti-*Trichinella* IgG antibodies were detected in all samples tested with the Home-ELISA, but in only four samples tested with the commercially-available kit. 1D-immunoblot analysis established a clear pattern of *T*. *spiralis* and *T*. *britovi* E-S ML proteins which reacted with *Trichinella*-infected human serum samples. Our findings indicate that *T*. *spiralis* and *T*. *britovi* ML E-S proteins demonstrated similar patterns of reactivity when blotted with representative serum samples from patients with the first clinical symptoms observed at 49 dpi. The most immunogenic proteins, i.e. those with highest intensity, were located in the region of 43–70 kDa and around 120 kDa. Several studies have evaluated the sensitivity and specificity of immunoblotting, and many of these have examined the main immunodominant proteins with diagnostic value, using E-S or muscle larvae extract [19,24–31]. Although most published papers use E-S antigens prepared according to the International Commission on Trichinellosis (ICT) protocol, the final product can vary; therefore, the level of recognition of the E-S protein fractions may differ between published studies. The molecular weights of the antigenic proteins observed in the present study, and the recognition of different protein fractions, differed from those identified in previous studies with regard to digestion protocol, age of larvae, the number of larvae per ml of basal medium, the purification method, E-S concentration, variation between human serum samples and detection method. Similarly, inter-study variation regarding the electrophoretic pattern and immunoblot could result from the fact that *T*. *britovi* demonstrates lower fecundity and immunogenicity than *T*. *spiralis* [32]. A similar specific banding pattern, characterized by bands around 125 kDa, and between 38 kDa to 78 kDa, were recognized by the IgG antibodies present in the sera from a *Trichinella*-infected patient in a 1D-immunoblot using *T*. *spiralis* E-S ML antigens by Pinelli et al. (2001) [31]. Recently, Gomez-Morales et al., (2018) compared immunogenic proteins from *T*. *spiralis* and *T*. *britovi* muscle larvae extract by 1D-immunoblotting [33]. Analysis of *T*. *spiralis*-infected human sera found *T*. *spiralis* and *T*. *britovi* antigens to demonstrate similar reactivity, and that the most immunogenic proteins with the highest intensity were located in the region between 50 and 75 kDa. Furthermore, human *T*. *britovi*-infected sera demonstrated the same pattern of reactivity as that of *T*. *spiralis*-infected patients with *T*. *spiralis* muscle larvae antigen.

An immunoproteomics approach including classic SDS-PAGE and 1D-immunoblot was also used to identify potentially early diagnostic proteins in *T*. *spiralis* E-S adult worms, which were recognized by the sera of patients with trichinellosis at 19 and 35 dpi [29]. Five immunogenic proteins bands around 55, 48–50, 45, 44, and 36 kDa were identified, and these were recognized by the sera from both patients. Following this, unique *T*. *spiralis* proteins were identified by mass spectrometry; of these, adult-specific DNase II, serine protease and serine protease inhibitor, are specific enzymes involved in parasite development, nutrition, host tissue invasion and immune evasion, and are considered as a new source of early diagnostics antigens for patients with trichinellosis [29].

Somboonpatarakun et al., (2018) [30] performed a comparative analysis using a similar proteomic approach to identify immunogenic proteins from somatic muscle larval extracts of three *Trichinella* species, including *T*. *spiralis*, *T*. *pseudospiralis* and *T*. *papuae*. Immunoblotting with sera pooled from ten adult patients infected with *T*. *spiralis* revealed the presence of specific protein bands located in the region from 33 to 67 kDa. Proteomic and bioinformatics analysis identified several immunogenic proteins (serine protease, actin-5C, intermediate filament protein ifa-1, deoxyribonuclease-2-alpha) involved in a great number of varied cellular and metabolic processes that contribute to the invasion of host tissue and larval molting [22].

The morphological and biological features of *T*. *britovi* are similar to those of *T*. *spiralis*; however, the proteomes of these two *Trichinella* species differ in molecular mass, antigenicity and peptide profiles [34]. The first comparative analysis of *T*. *spiralis* and *T*. *britovi* ML E-S proteins was performed by a combination of two-dimensional difference electrophoresis (2-D DIGE), immunoblotting and mass spectrometry [24]. It was concluded that 2-D DIGE and 2-DE immunoblotting approaches showed that both *Trichinella* species produce somewhat different immunoproteomes; these included both species-specific and common proteins which reacted positively against sera from pigs experimentally infected with *T*. *spiralis* or *T*. *britovi*. The common proteins were identified as gp43 glycoprotein and different variants of serine-proteases. *T*. *britovi*-specific proteins included 5'-nucleotidase isoforms [24]. The antigenic differences of both *Trichinella* species analyzed by 2-D immunoblotting demonstrated that E-S proteins might be used as species-specific diagnostic markers of *Trichinella* infection.

Our 2D-immunoblot findings show that 22 (*T*. *spiralis*) and 18 (*T*. *britovi*) protein spots with 50–60 kDa were recognized by human sera at 49 dpi and successfully identified by LC-MS/MS. Of these proteins, the following were common to both *Trichinella* species: serine protease 9, serine protease, antigen targeted by protective antibodies and Actin-1, partial. Hypothetical protein T01_7775 and P49 antigen, partial was specific to *T*. *spiralis* while deoxyribonuclease-2-alpha was typical of *T*. *britovi*. Our present findings reveal the presence of a range of proteins known to be involved in the mechanisms of host cells and also play roles in tissue invasion, larval migration or molting, immune modulation and metabolic processes in other helminths.

GO analysis found the identified proteins to participate in hydrolytic processes. Transmembrane protease serine 9 and serine protease, which were characteristic for both *T*. *spiralis* and *T*. *britovi* ML E-S antigens, possess serine-type endopeptidase activity and serine-type peptidase activity. Additionally, transmembrane protease serine 9 and one uncharacterized protein (hypothetical protein T12_16967) serve as integral components of cellular membranes. In addition, both the P49 antigen identified in *T*. *spiralis* ML E-S and deoxyribonuclease-2-alpha identified in *T*. *britovi* ML E-S have the same deoxyribonuclease II activity and they have been found to exert an important role in pathogen invasion in evading host defense [35]. Another antigen targeted by protective antibodies (AAA20539.1), though not assigned a GO-molecular function, demonstrates 99% similarity in their amino acid sequence with the chymotrypsin-like protease (AKE78867.2). Although the actin-1 protein identified in the extracts could not be assigned any verified molecular function, its sequence similarities to the actin family proteins suggests that it may operate as an ATP binding protein. The BLAST analysis of the hypothetical proteins from *T*. *britovi* sequences (hypothetical protein T03_17187; KRY50178.1) and T12_7360; KRY12204.1) demonstrates above 90% similarity with the multi-cystatin-like domain protein precursor (CBX25716.1).

Some of the identified in presented study proteins were also previously found in other life stages of *Trichinella* as highly represented and are considered to be potentially diagnostic antigens and vaccine candidates for trichinellosis. The antigen targeted by protective antibodies (AAA20539.1) is the same *Trichinella* protein as the 31 kDa protein in *T*. *spiralis* (Ts31, Genbank: U01847.1). The Ts31 protein contains a trypsin-like serine protease domain which facilitates *T*. *spiralis* invasion of the intestinal epithelium, which could make it a vaccine target candidate against *Trichinella* infection [36].

Enzymes with the deoxyribonuclease-2 activity (DNase II) including P49 antigen and deoxyribonuclease-2-alpha could play a critical role in larval invasion, development, survival of the parasite as well as immunodiagnosis [26,28,30,37–39]. The partial P49 antigen successfully identified in the *T*. *spiralis* ML E-S proteome had previously been characterized and expressed in *Escherichia coli* as a potentially valuable antigen both for vaccine development and immunodiagnosis by Su et al., (1991) [37]. The presence of deoxyribonuclease-2-alpha (DNase II) in our study was only observed in the *T*. *britovi* ML E-S proteome against human sera; however, it was also successfully identified in the proteomes of *T*. *britovi* and *T*. *spiralis* somatic muscle larvae when pig sera (60 dpi) and human sera were used, respectively [19,30]. Comparative analysis of protein sequence showed that half of *Trichinella* spp DNase II genes encode E-S products which participate in the host-parasite interactions. Thereby, these proteins might be considered as early diagnostic markers of trichinellosis, as well as vaccine candidates for its prevention [26,40,41].

A 1D-immunoblot and LC-MS/MS-based study of *Trichinella* by Somboonpatarakun et al. (2018) identified the protein DNAase II among the immunoproteins recognized by *T*. *spiralis*–infected human sera; the protein was also found to be common between the proteomes of *T*. *spiralis*, *T*. *pseudospiralis* and *T*. *papuae* muscle larvae [30]. A comparison of the immunogenicity of infective muscle larvae of *T*. *spiralis*, *T*. *pseudospiralis* and *T*. *papuae* found that some of their proteins, *viz*. serine protease, deoxyribonuclease-2-alpha and 5’-nucleotidase, play a role in inducing the key immune response in humans and might be used to improve serological diagnosis [30]. Liu et al., (2016) report that among the immunogenic proteins from adult worm E-S, *T*. *spiralis* adult-specific DNAase II was recognized by early infection sera from infected mice at 8 dpi, suggesting that this protein may have diagnostic potential [42].

Serine proteases with chymotrypsin-like, elastase-like or trypsin-like activities are expressed by different stages of *Trichinella*, and these are common proteins recognized by *Trichinella*-infected human serum in the ML E-S of both *T*. *spiralis* and *T*. *britovi* [29,39,42–44]. This proteins play an important part in physiological and pathological processes during parasite infection: in *Trichinella* infection, serine protease facilitate larval invasion, molting, digestion, fibrinolysis and help the parasite evade the host immune response [24,43,45].

Comparative 1D-immunoblot analysis of the proteomes of three *Trichinella* species (*T*. *spiralis*, *T*. *pseudospiralis*, *T*. *papuae*) using *T*. *spiralis*- infected serum samples found serine protease to be the main immunodominant protein common to *T*. *spiralis* and *T*. *papuae*, and that it may be a suitable vaccine candidate or diagnostic antigen [30].

A recent report described the expression of recombinant serine proteinase (rTsSP) in *Escherichia coli* as a potentially early diagnostic antigen for human trichinellosis [44] and a potential target for vaccines against enteral *Trichinella* infection [46]. To investigate the potential use of rTsSP for serodiagnosis of human trichinellosis, ELISA tests based on rTsSP and ML E-S were applied to detect antibodies in serum samples with trichinellosis, with the two tests demonstrating high sensitivity: 98.11% for rTsSP-ELISA and 88.68% for ES-ELISA. The sensitivity of both antigens reached 100% when the patient serum samples at 35 dpi were tested; however, the sensitivity of rTsSP-ELISA was significantly greater than ES-ELISA when testing *Trichinella*-infected human sera at 19 dpi [44]. This finding emphasizes the value of rTsSP protein as a serodiagnostic tool for human trichinellosis.

The recombinant serine proteinase (rTsSPI) from *T*. *spiralis* adult worms has also been identified as a novel potential target for anti-*Trichinella* vaccine [47]. Vaccination of mice with rTsSPI triggered a strong anti-TsSPI IgG response; in addition, intestinal adult worm recovery identified a 62.2% reduction in burden at six days post-infection (dpi) and a 57.25% reduction in ML at 35 dpi [47]. In addition, the recombinant adult *T*. *spiralis* serine protease (rTsSP-ZH68) was recognized by the sera of infected mice at 8–10 dpi and the sera of early patients with trichinellosis at 19 dpi; the protein has been identified as a promising candidate for the early diagnosis of trichinellosis and as a potential vaccine [29].

The multi-cystatin-like domain protein (CLP) which had above 90% identity in their amino acid sequence to two identified hypothetical *T*. *britovi* proteins (hypothetical protein T03_17187; T12_7360) is promising immunoreactive protein used in recombinant form to control trichinellosis [48–50]. A previous study by Tang et al. 2015 [49] showed that recombinant protein of *T*. *spiralis* (rTs-CLP) was recognized by pig antiserum as early as 15 dpi (pigs infected with 20 000 ML of *T*. *spiralis)* and could induce protective immunity in mice, with a 61.21% reduction in the number of muscle larvae. An alternative study by Stachyra et al., 2019 [50] with a recombinant protein of *T*. *britovi* (rTb-CLP) identified seroconversion at 24 dpi when sera from pigs experimentally infected with 5.000 ML *T*. *britovi* was used, while a 46.9% reduction in ML worm burden was observed in mice immunized with rTb-CLP protein. The diagnostic value of CLP protein was confirmed also by Liu et al. (2016) using a proteomic approach. CLP protein was successfully recognized in the adult worm E-S proteome by the sera of mice infected with *T*. *spiralis* at 8 dpi [42].

The main immunogenic proteins recognized in the present study by the sera of patients at 49 dpi, both those common to *T*. *spiralis* and *T*. *britovi* ML E-S and those that are only specific to either species, may offer potential for use in vaccine development. They may hence be considered as a solution to improve diagnostic antigens for trichinellosis, as well as to differentiate *Trichinella s*pecies serologically.

The proteins identified in the proteomes of ML E-S *T*. *spiralis* and *T*. *britovi* displayed conserved epitopes recognized by antibodies in *Trichinella*-infected human sera. As *Trichinella*-infected human sera (infected as *T*. *spiralis*) recognized the same immunogenic proteins in both encapsulated (*T*. *spiralis* and *T*. *britovi*) and non-encapsulated species (*T*. *pseudospiralis* and *T*. *papuae*), it is possible that proteins from *Trichinella* species other than *T*. *spiralis* may be used as target antigens for the detection of *Trichinella* infection.

Therefore, further information about both *Trichinella* species-specific and common E-S antigens is required to support the development of species-specific diagnostics.

## Conclusions

Both the 2DE-electrophoresis and the 2D-immunoblotting approaches indicate that *T*. *spiralis* and *T*. *britovi* produce partially distinctive antigen profiles, which contain E-S proteins that offer potential as species-specific diagnostic markers for *Trichinella* infection. Our results demonstrate also the value of proteomic analysis as a versatile tool for comparing the secretomes of different *Trichinella* species, and to identify the factors which contribute to the interaction with the host.

To the best of our knowledge, this is the first proteomic study using 2D-immunoblot to focus on *T*. *spiralis* and *T*. *britovi* ML E-S specific immunoreactive proteins recognized by human *Trichinella*-infected sera.

## Supporting information

S1 Raw images (PDF)
