# Supplementary material for: Immunoproteomic analysis of Trichinella spiralis and Trichinella britovi excretory-secretory muscle larvae proteins recognized by sera from humans infected with Trichinella
Source: PLoS One. 2020 Nov 5;15(11):e0241918. doi: 10.1371/journal.pone.0241918 (PMC7644068; doi:10.1371/journal.pone.0241918)

Supporting Information

All of the gels and films images were captured with a ChemiDoc MP system (BioRad, USA). PageRurel Plus Prestained Protein Ladder, 10 to 250 kDa (ThermoFisher Scientific, Walthman, USA) was used as molecular weight marker for protein separation on each gel and Western blotting. Panels marked with red boxes on raw images corresponds to areas which were used for final figures generation.

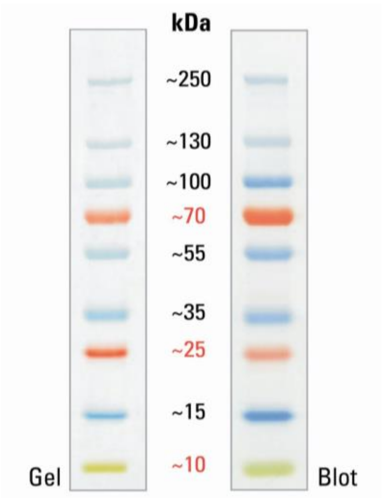

PageRurel Plus Prestained Protein Ladder
(ThermoFisher Scientific, Walthman, USA)

Figure 1_ Raw Image

The raw image of the silver stained SDS-PAGE gel with loaded muscle larvae (ML) excretory-secretory proteins of *T. spiralis* (E-S T1) and *T. britovi* (E-S T3).

Detection: PlusOne Silver Staining Kit (GE Healthcare)

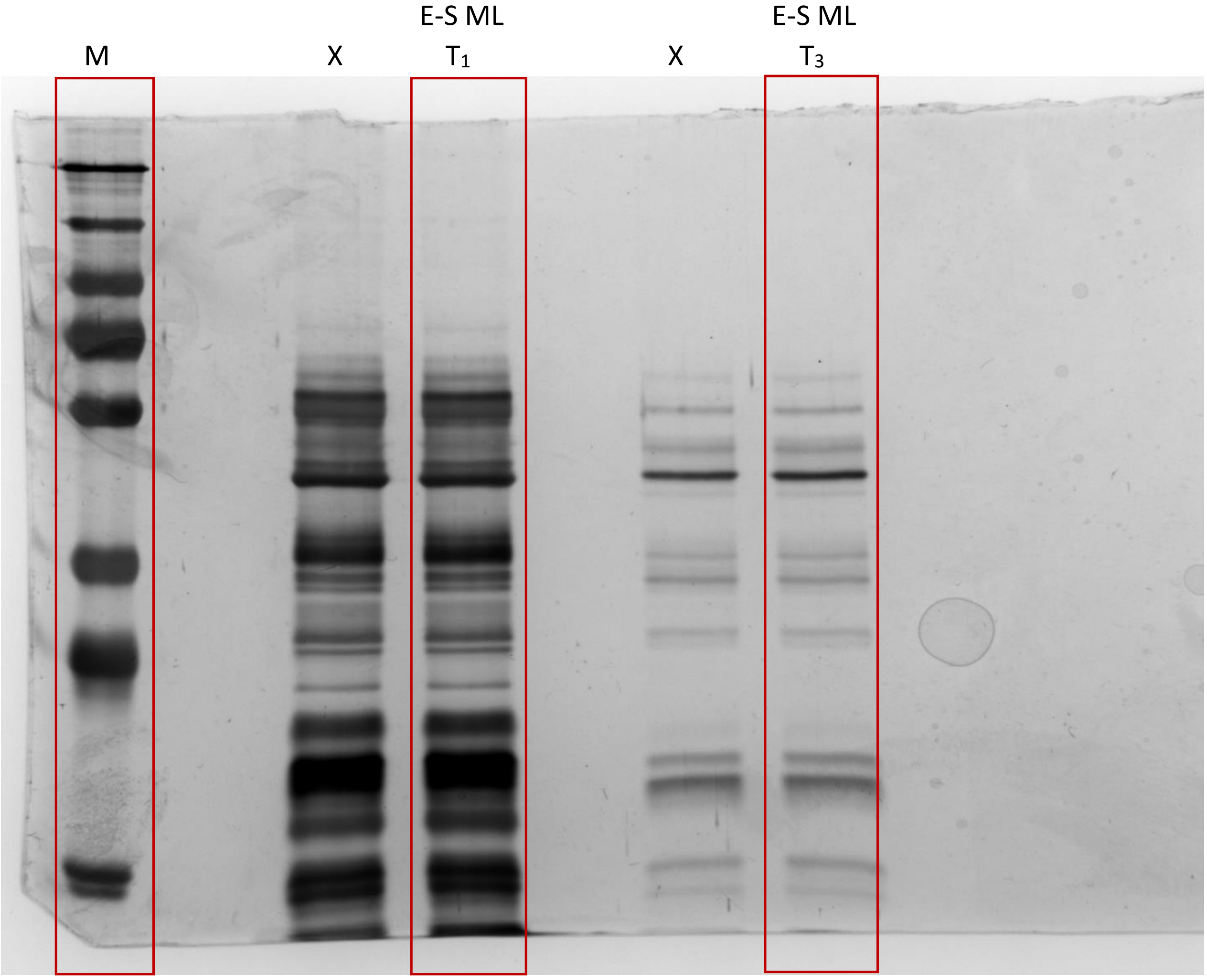

Figure 2A_ Raw Image

The raw image of the immunoblot of *T. spiralis* muscle larvae excretory-secretory proteins incubated with *Trichinella*-infected human sera samples and the negative control samples. Marked in red lane 6 was used for analysis of bands signal intensity and relative migration. The numbers shows the order in which membrane strips are presented on Fig. 2A.

Detection: SIGMAFAST™ 3,3′-Diaminobenzidine (Sigma-Aldrich, Louis, USA).

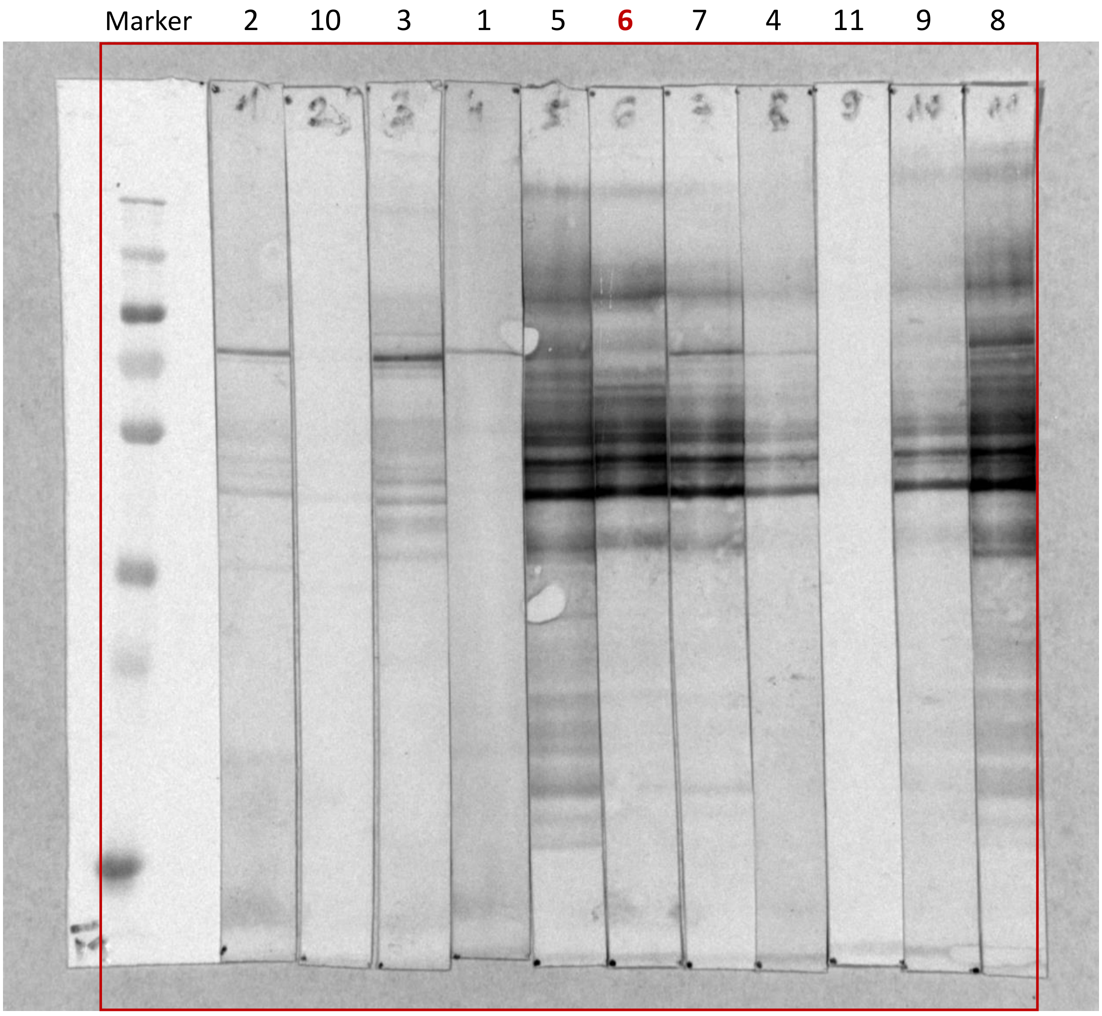

Figure 2B_ Raw Image

The raw image of the immunoblot of *T. britovi* muscle larvae excretory-secretory proteins incubated with *Trichinella*-infected human sera samples and the negative control samples. Marked in red lane 5 was used for analysis of bands signal intensity and relative migration. The numbers shows the order in which membrane strips are presented on Fig. 2B.

Detection: SIGMAFAST™ 3,3′-Diaminobenzidine (Sigma-Aldrich, Louis, USA).

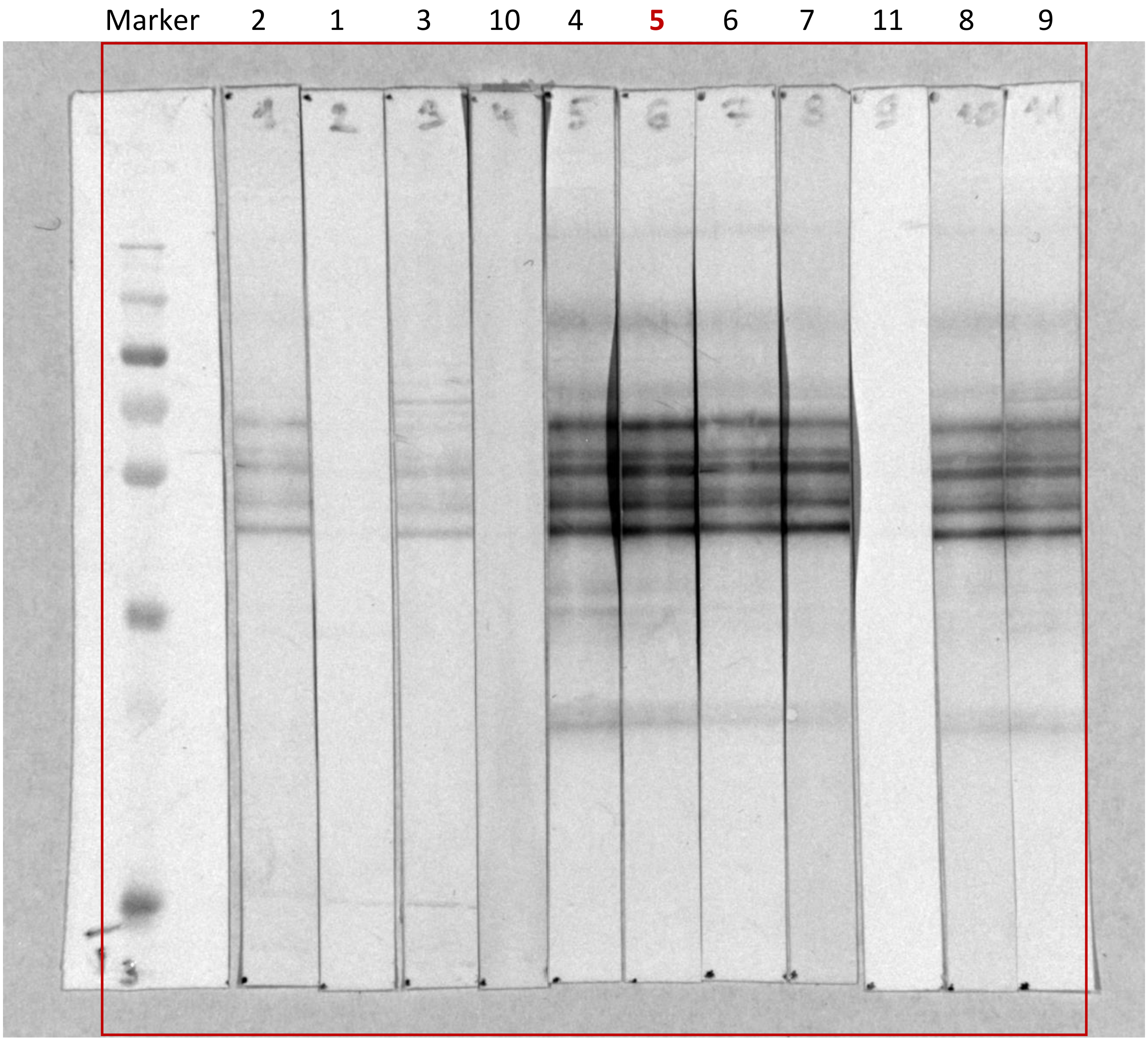

Figure 3A_ Raw Image

The raw image of the silver stained 2-DE gel with separated *T. spiralis* muscle larvae excretory-secretory proteins.

Detection: PlusOne Silver Staining Kit (GE Healthcare).

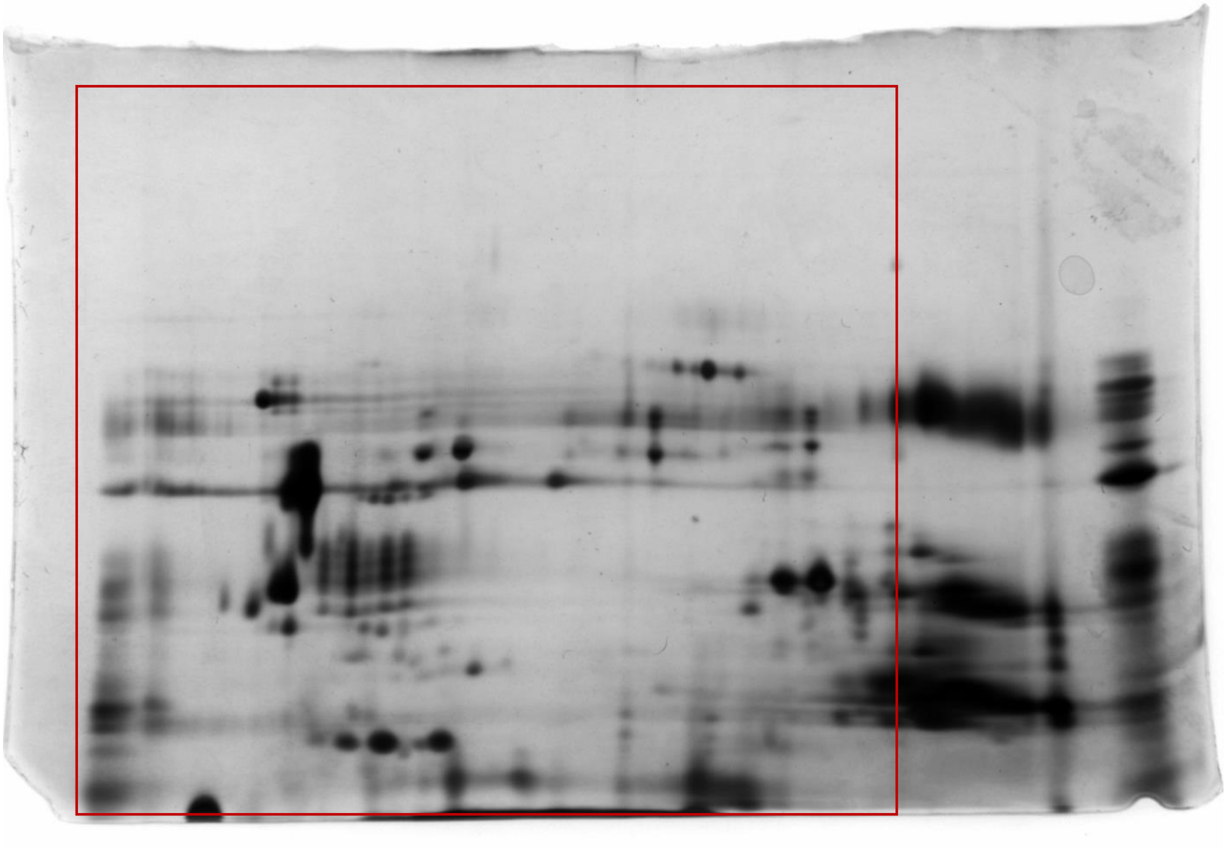

Figure 3B_Raw Image

An image of 2D-immunoblot of *T. spiralis* muscle larvae excretory-secretory proteins incubated with *Trichinella*-infected human sera sample at 14 days post infection (dpi).

Detection: Super Signal West Pico Chemiluminescent Substrate (ThermoFisher Scientific, Walthman, USA), visualized on a film.

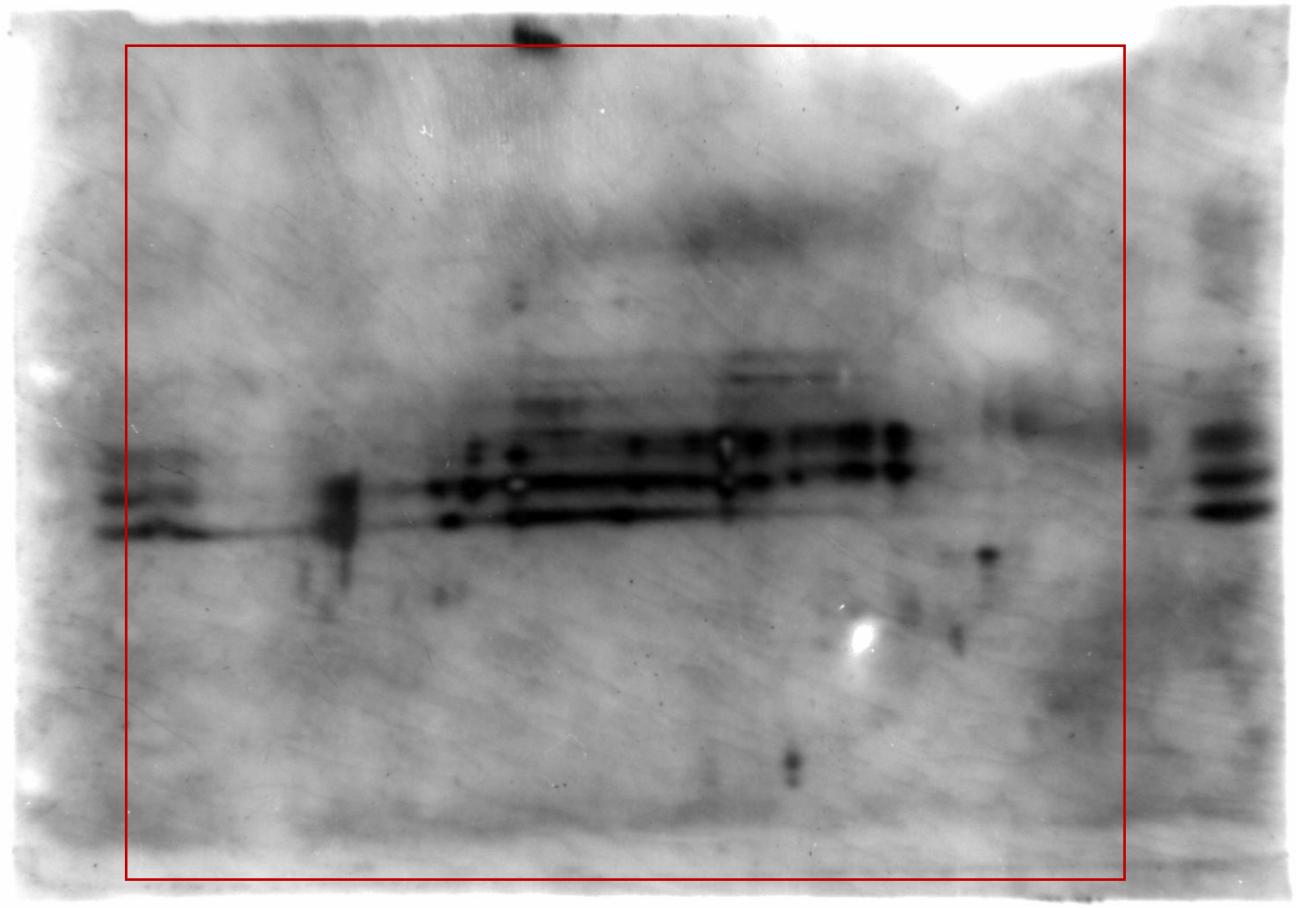

Figure 3C_ Raw Image

The raw image of silver stained 2-DE gel with separated *T. britovi* muscle larvae excretory-secretory proteins.

Detection: PlusOne Silver Staining Kit (GE Healthcare)

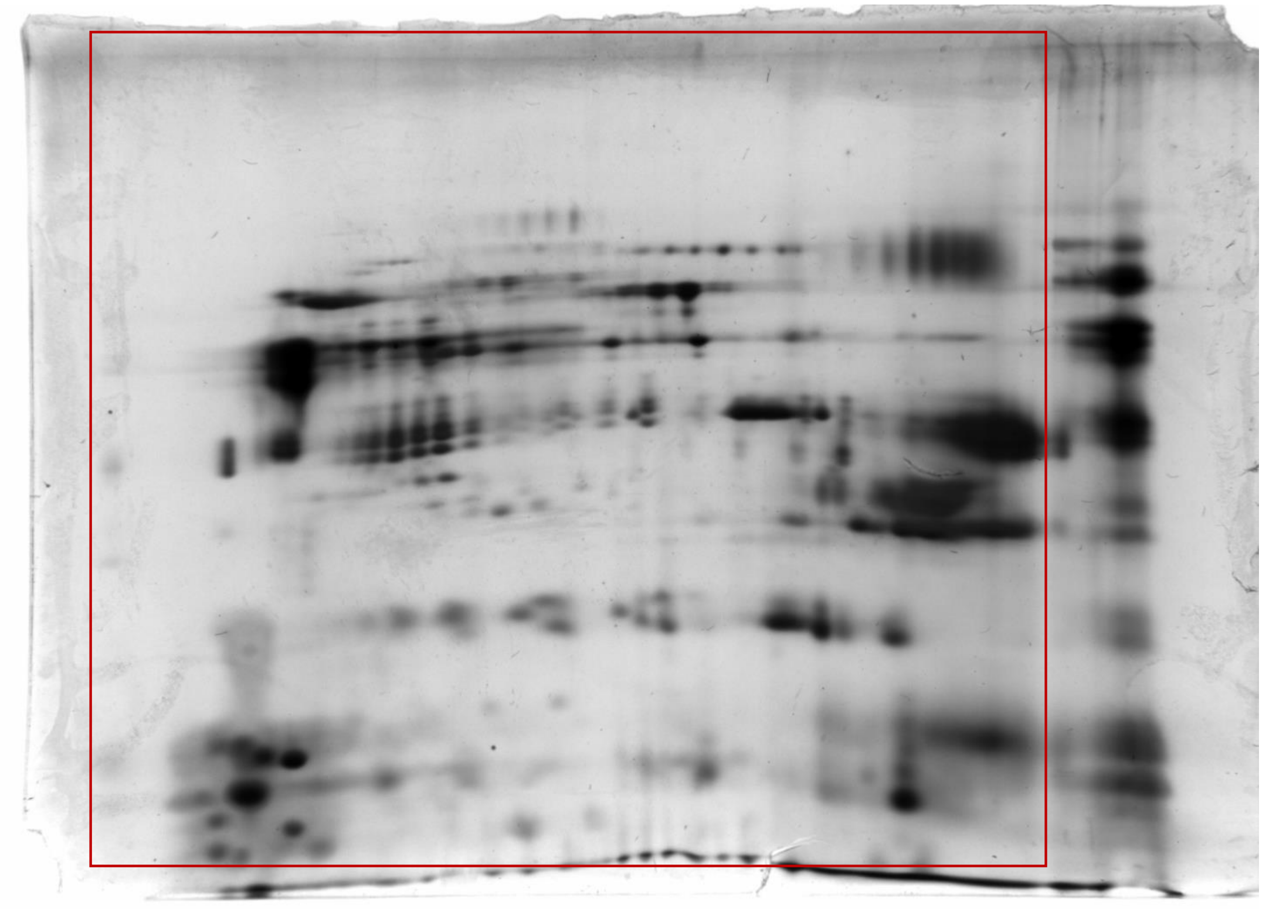

Figure 3D_ Raw Image

An image of 2D-immunoblot of *T. britovi* muscle larvae excretory-secretory proteins incubated with *Trichinella*-infected human sera sample at 14 days post infection (dpi).

Detection: Super Signal West Pico Chemiluminescent Substrate (ThermoFisher Scientific, Walthman, USA), visualized on a film.

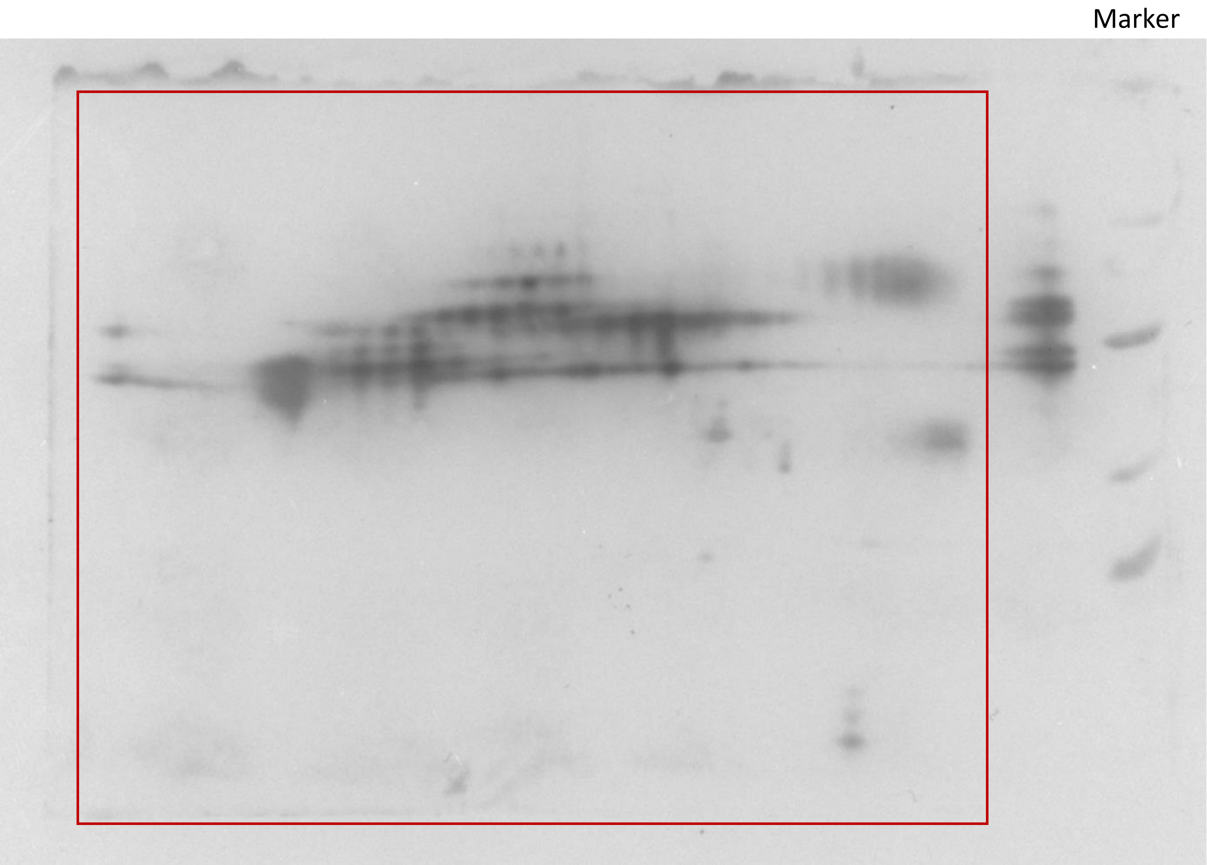

Supplement: S1 Raw images — (PDF) [file pone.0241918.s001.pdf]
